# Cement-augmented pedicle screw for thoracolumbar degenerative diseases with osteoporosis: a systematic review and meta-analysis

**DOI:** 10.1186/s13018-023-04077-w

**Published:** 2023-08-28

**Authors:** Zhoufeng Song, Qiujun Zhou, Xiaoliang Jin, Jinjie Zhang

**Affiliations:** 1 The First Affiliated Hospital of Zhejiang Chinese Medical University (Zhejiang Provincial Hospital of Chinese Medicine), 54 Post and Telegraph Road Uptown, Hangzhou, 310000 China; 2 Department of First Clinical Medical College, Zhejiang Chinese Medical University, Hangzhou, 310000 China

**Keywords:** Cement-augmented, Pedicle screw, Internal fixation, Osteoporosis, Thoracolumbar degenerative disease, Systematic review, Meta-analysis

## Abstract

**Background:**

Cement-augmentation pedicle screws have been widely used in spinal internal fixation surgery combined with osteoporosis in recent years, which can significantly improve the fixation strength, but compared with conventional methods, whether it has more advantages is still inconclusive of evidencebased medicine. To systematically evaluate the efficacy and safety of cement-augmented pedicle screw in the treatment of thoracolumbar degenerative diseases with osteoporosis.

**Methods:**

We searched PubMed, Embase, and Cochrane Library for studies published from the establishment of the database up until June 2023. We included studies that concerning the cement-augmented pedicle screw and the traditional pedicle screw placement for thoracolumbar degenerative diseases with osteoporosis. We excluded repeated publication, researches without full text, incomplete information or inability to conduct data extraction and animal experiments, case report, reviews and systematic reviews. STATA 15.1 software was used to analyze the data.

**Results:**

A total of 12 studies were included in this meta-analysis. The sample size of patients were totally 881, of which, 492 patients in cement-augmented screw group and 389 patients in conventional screw group. Meta-analysis results showed that Japanese Orthopaedic Association (JOA) score (WMD = 1.69, 95% CI 1.15 to 2.22), intervertebral space height (WMD = 1.66, 95% CI 1.03 to 2.29) and post-operation fusion rate (OR = 2.80, 95% CI 1.49 to 5.25) were higher in the cement-augmented screw group than those in the conventional screw group. Operation time was longer in the cement-augmented screw group than that in the conventional screw group (WMD = 15.47, 95% CI 1.25 to 29.70). Screw loosening rate was lower in the cement-augmented screw group than those in the conventional screw group (OR = 0.13, 95% CI 0.07 to 0.22). However, hospitalization time, intraoperative blood loss and Visual analog scale (VAS) score were not significantly different between the two groups (*P* > 0.05).

**Conclusion:**

Compared with conventional pedicle screw placement, cement-augmented pedicle screw is more effective in the treatment of osteoporotic thoracolumbar degenerative disease by improving fusion rate and interbody height, reducing the incidence of screw loosening, and elevating long-term efficacy.

**Supplementary Information:**

The online version contains supplementary material available at 10.1186/s13018-023-04077-w.

## Introduction

Thoracolumbar degenerative diseases are common diseases in middle-aged and elderly people, which often cause patients with low back pain, lower extremity pain and limited movement. Finally, the pedicle screw system is required for internal fixation to improve spinal stability and alleviate symptoms, which brings serious mental and economic burden to patients [1, 2] With the development of modern medicine, the average life expectancy of residents in the world continues to increase, the aging population is increasing, and the population suffering from osteoporosis is becoming more and more common, and people in this age group are often accompanied by serious thoracic and lumbar degenerative diseases [3, 4]. When the traditional pedicle screw is inserted in patients with osteoporosis, the screw is often easy to loosen due to the poor bone mineral density, resulting in the failure of internal fixation [5]. Therefore, how to effectively use pedicle screws to reconstruct the spine of patients with thoracolumbar degenerative diseases, column stability, and avoid the failure of internal fixation due to nail loosening, nail extraction and other problems has been a research focus of spinal surgeons.

With the development of science and technology, spinal surgery techniques have been continuously improved, among which methods to improve the stability of pedicle screws have also been increasing. At present, three methods are mainly used: (1) Using bone cement to strengthen pedicle screws [6]; (2) Increase the contact area between screws and bone cortex, such as cortical bone screws and double-threaded screws [7]; (3) Modify the screws themselves, such as expansion screws with increased diameters and hydroxy-phosphate-lime coated screws [8, 9].

Cement-augmented pedicle screw technology is one of the common techniques to improve the stability of internal fixation of pedicle screw. Compared with traditional pedicle screw fixation, this technique can effectively improve the success and rate of internal fixation. However, the comparison of the clinical efficacy of the treatment of osteoporotic thoracic and lumbar degenerative diseases is still lack of evidence-based medical conclusion [10]. In this study, a clinical comparative study of bone cement-strengthened pedicle screw fixation and traditional pedicle screw fixation in the treatment of thoracolumbar degenerative diseases was collected, and a meta-analysis was conducted after strict screening, in order to provide reference for the fusion fixation of thoracolumbar degenerative diseases in clinic.

## Methods

### Literature inclusion and exclusion criteria

Inclusion criteria: the study type is retrospective or prospective study; studies that reports the cement-augmented pedicle screw and the traditional pedicle screw placement for thoracolumbar degenerative diseases with osteoporosis; the language is limited to English.

Exclusion criteria: repeated publication; studies without full text, incomplete information or inability to conduct data extraction; animal experiments; case report; reviews and systematic reviews.

### Search strategy

In this meta-analysis, we searched Pubmed, Embase, Cochrane Library from establishment of the database to June, 2023. The search terms are as follows: (“osteoporosis”) AND (“pedicle screw” OR “fixation”) AND (“cement” OR “polymethyl methacrylate” OR “PMMA” OR “augmentation”).

### Literature screening and data extraction

Two researchers independently carried out literature search, screening and information extraction. When a question or dispute arises, a decision is made after discussion or negotiation with a third person. The data extraction included the author, publication year of articles, study design, sample size, age, sex and outcomes including hospitalization time (day), operation time (minute), intraoperative blood loss (ml), Japanese Orthopaedic Association (JOA) score, Visual analog scale (VAS) score, intervertebral space height, fusion rate and screw loosening rate.

### Literature quality assessment

The Newcastle–Ottawa Scale (NOS) for evaluating the quality of published literature is carried out separately by two academics [11], and it was used to evaluate the quality of 16 cohort studies, NOS includes 4 items (4 points) for “Research Subject Selection”, 1 item (2 points) for “Comparability between Groups” and 3 items (3 points) for “Result Measurement”, with a full score of 9 points and ≥ 7 is regarded as High-quality literature, < 7 is divided into lower-quality literature.

### Data synthesis and statistical analysis

All data analyzed by STATA 15.1 (Stata Crop LP, College Station, TX) [12]. Weighted mean difference (WMD) (90%CI) is used to evaluate continuity variables. *I*^2^ and *Q* test were used to evaluate heterogeneity. If the heterogeneity test is* P* ≥ 0.1 and *I*^2^ ≤ 50%, it indicates that there is homogeneity between studies, and the fixed effects model is used for combined analysis; if *P* < 0.1 or *I*^2^ > 50%, it indicates that there is heterogeneity and sensitivity analysis was used to find the source of heterogeneity. If the heterogeneity is still large, use the random effects model or give up the combination of results and use descriptive analysis. Funnel plot was used to assess the publication bias.

## Results

### The results of literature search

**Fig. 1:**
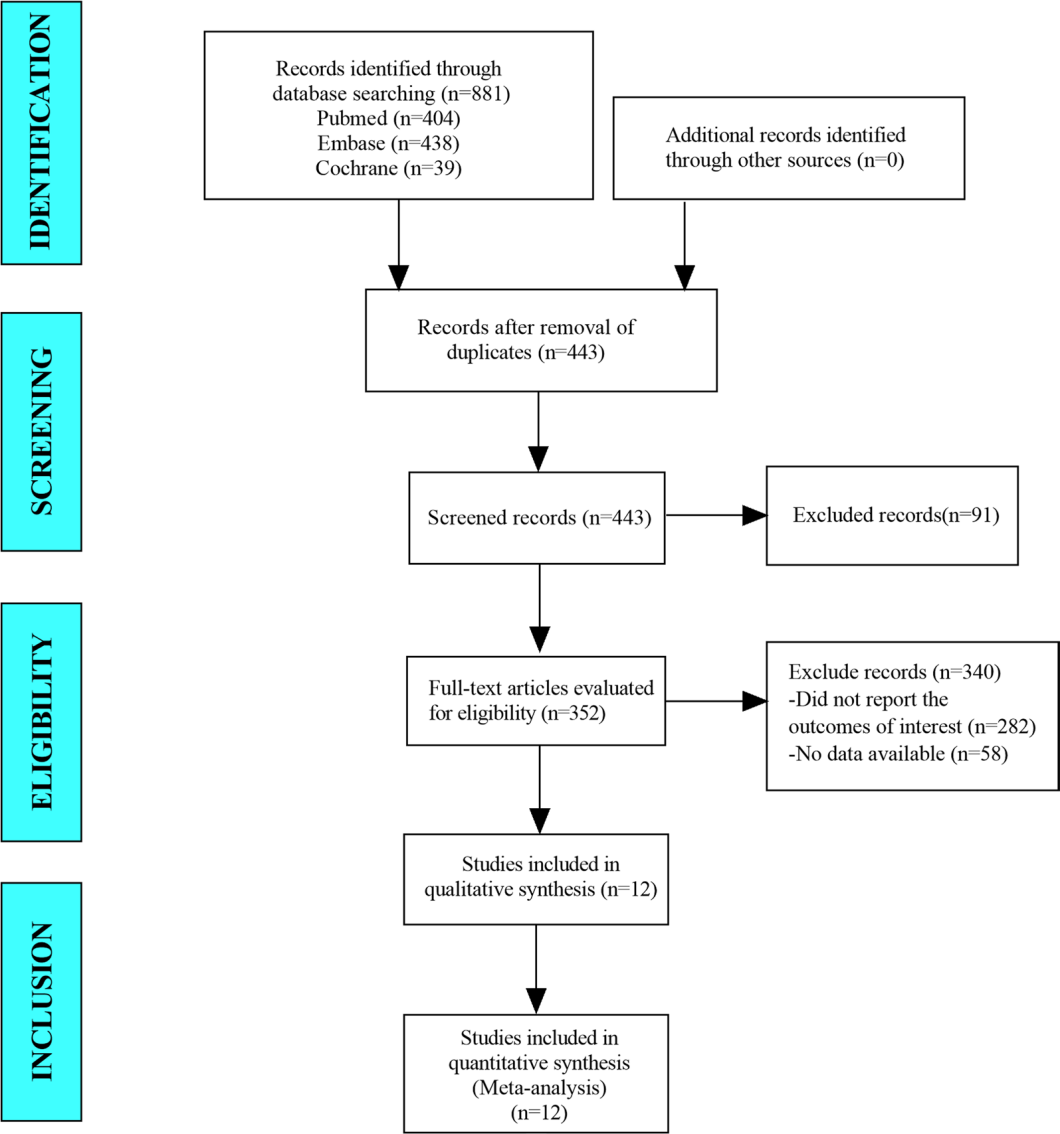
Flow diagram for selection of studies

In this meta-analysis, a total of 763 studies were retrieved from the database including Pubmed, Embase and Cochrane Library. After eliminating duplicate studies, 394 were obtained. After browsing titles and abstracts, 123 studies were obtained. Finally, 6 articles were included in the meta-analysis (Fig. 1).

### Baseline characteristics and quality assessment of the included studies

A total of 12 studies were included in this meta-analysis. The sample size of patients were totally 881, of which, 492 patients in cement-augmented screw group and 389 patients in conventional screw group. The average age in cement-augmented screw group ranged from 49.0 to 76.0, while the conventional screw group ranged from 48.0 to 75.0. Average bone density ranged from − 2.5 to − 3.3 in cement-augmented screw group and − 2.5 to − 3.2 in conventional screw group. NOS scores were all above 8 points, indicating the literature included is of moderate or high quality (Table 1).

**Table 1:** Baseline characteristics and quality assessment of the included studies

Author	Year	Study design	Sample size		Sex		Age		Bone density		Follow-up time (Year)		NOS score	Bone cement reinforcement method
			Cement-augmented screw	Conventional screw	Cement-augmented screw	Conventional screw	Cement-augmented screw	Conventional screw	Cement-augmented screw	Conventional screw	Cement-augmented screw	Conventional screw		
Kim et al. [13]	2010	Cohort study	31	31	1/30	2/29	49.0–77.0	48.0–72.0	− 2.5 ~ − 4.7	− 2.5 ~ − 4.5	25.0–58.0	25.0–42.0	8	Conventional pedicle screws
Xie et al. [14]	2011	Cohort study	14	17	1/13	5/12	63.1 ± 9.0	59.0 ± 6.6	− 3.0 ± 0.4	− 3.1 ± 0.3	> 24.0	8	Conventional pedicle screws	
Sawakami et al. [15]	2011	Cohort study	17	21	5/12	6/15	73.6 ± 1.6	73.5 ± 1.8	–	–	33.6 ± 6.6	28.6 ± 3.5	9	Conventional pedicle screws
Seo et al. [16]	2012	Cohort study	157	93	49/108	37/56	49.0–74.0	46.0–69.0	− 3.9	− 3.6	10.0–23.0	–	9	Conventional pedicle screws
El Saman et al. [17]	2013	Cohort study	15	9	4/11	4/5	76.0 ± 9.3	75.0 ± 8.9	–	–	0.12–4.0	9	Conventional pedicle screws	
Sun et al. [18]	2016	Cohort study	14	12	2/12	1/11	67.1 ± 9.2	70.7 ± 6.6	− 3.2 ± 0.6	− 3.1 ± 0.5	10.6 ± 2.3	36.5 ± 7.2	8	Fenestrated pedicle screws
Sun et al. [19]	2017	Cohort study	15	19	1/14	5/14	69.2 ± 6.6	69.0 ± 8.5	− 2.7 ± 0.1	− 2.7 ± 0.1	12.0	8	Fenestrated pedicle screws	
Cao et al. [20]	2018	Cohort study	23	24	7/16	11/13	66.9 ± 7.1	65.3 ± 5.2	− 3.2 ± 0.4	− 3.1 ± 0.5	24.0	8	Conventional pedicle screws	
Wang et al. [21]	2019	Cohort study	36	52	2/34	12/40	65.9 ± 9.4	67.3 ± 6.6	− 3.3 ± 0.5	− 3.2 ± 0.5	15.4 ± 6.6	36.6 ± 5.9	8	Fenestrated pedicle screws
Mo et al. [22]	2019	Cohort study	28	28	2/26	3/25	67.1 ± 1.3	66.0 ± 1.1	− 3.0 ± 0.2	− 3.0 ± 0.1	35.0 ± 7.4	33.6 ± 5.4	8	Fenestrated pedicle screws
Kim et al. [23]	2020	Cohort study	96	36	18/78	10/26	73.0 ± 6.2	74.2 ± 5.7	–	–	> 24.0	9	Conventional pedicle screws	
Tang et al. [24]	2022	Cohort study	46	47	7/39	6/41	70.7 ± 7.2	67.9 ± 7.6	− 3.2 ± 0.9	− 2.9 ± 0.5	31.9 ± 15.5	35.5 ± 21.5	8	Conventional pedicle screws

### Results of the meta-analysis

#### Hospitalization time (day)

**Fig. 2:**
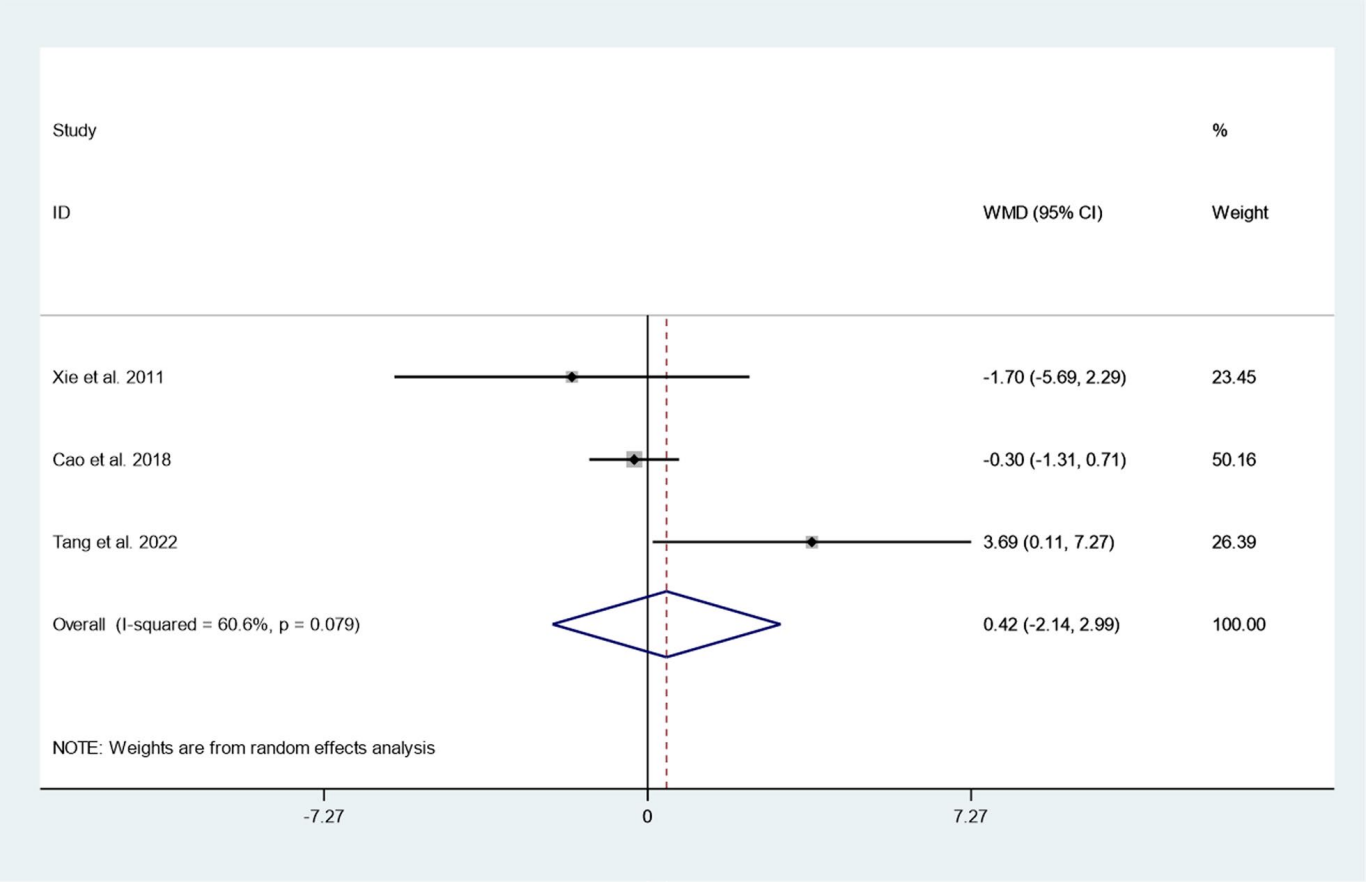
Forest plot of hospitalization time between cement augmented screw group and conventional screw group

Three articles reported hospitalization time between cement augmented screw group and conventional screw group. Since there was heterogeneity in the study, the random-effect model was used to combine the effect sizes (*I*^*2*^ = 60.6%*, P* = 0.079). The pooled results showed that the difference in hospitalization time between the cementaugmented screw group and the conventional screw group was not statistically significant (WMD = 0.42, 95% CI − 2.14 to 2.99; P = 0.745) (Fig. 2).

#### Operation time (minute)

**Fig. 3:**
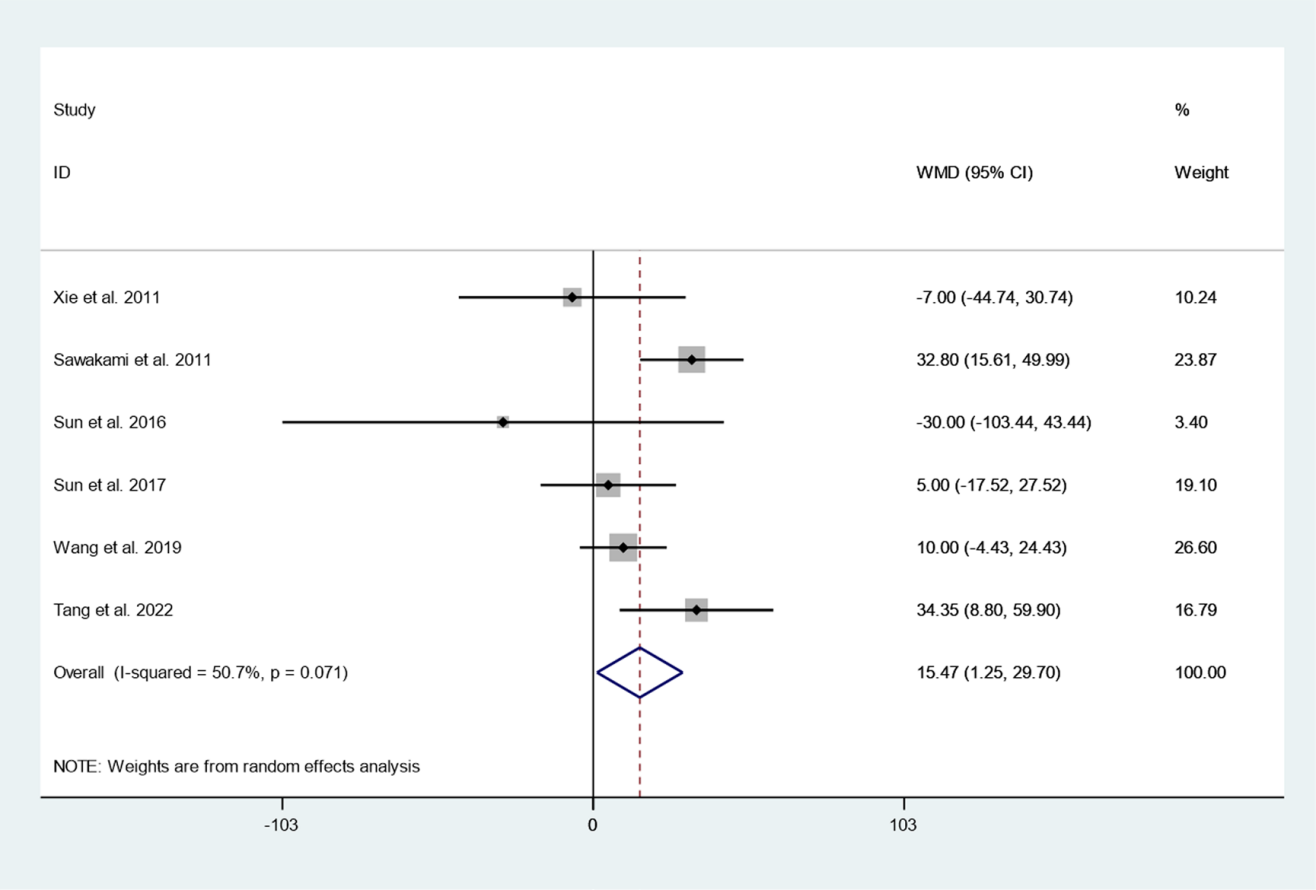
Forest plot of operation time between cement augmented screw group and conventional screw group

Six articles reported operation time between cement augmented screw group and conventional screw group. Since there was heterogeneity in the study, the random-effect model was used to combine the effect sizes (*I*^*2*^ = 50.7%, *P* = 0.071). The pooled results show that the operation time of the cement augmented screw group was significantly longer than that of the conventional screw group (WMD = 15.47, 95% CI 1.25 to 29.70; *P* = 0.033) (Fig. 3).

#### Intraoperative blood loss

**Fig. 4:**
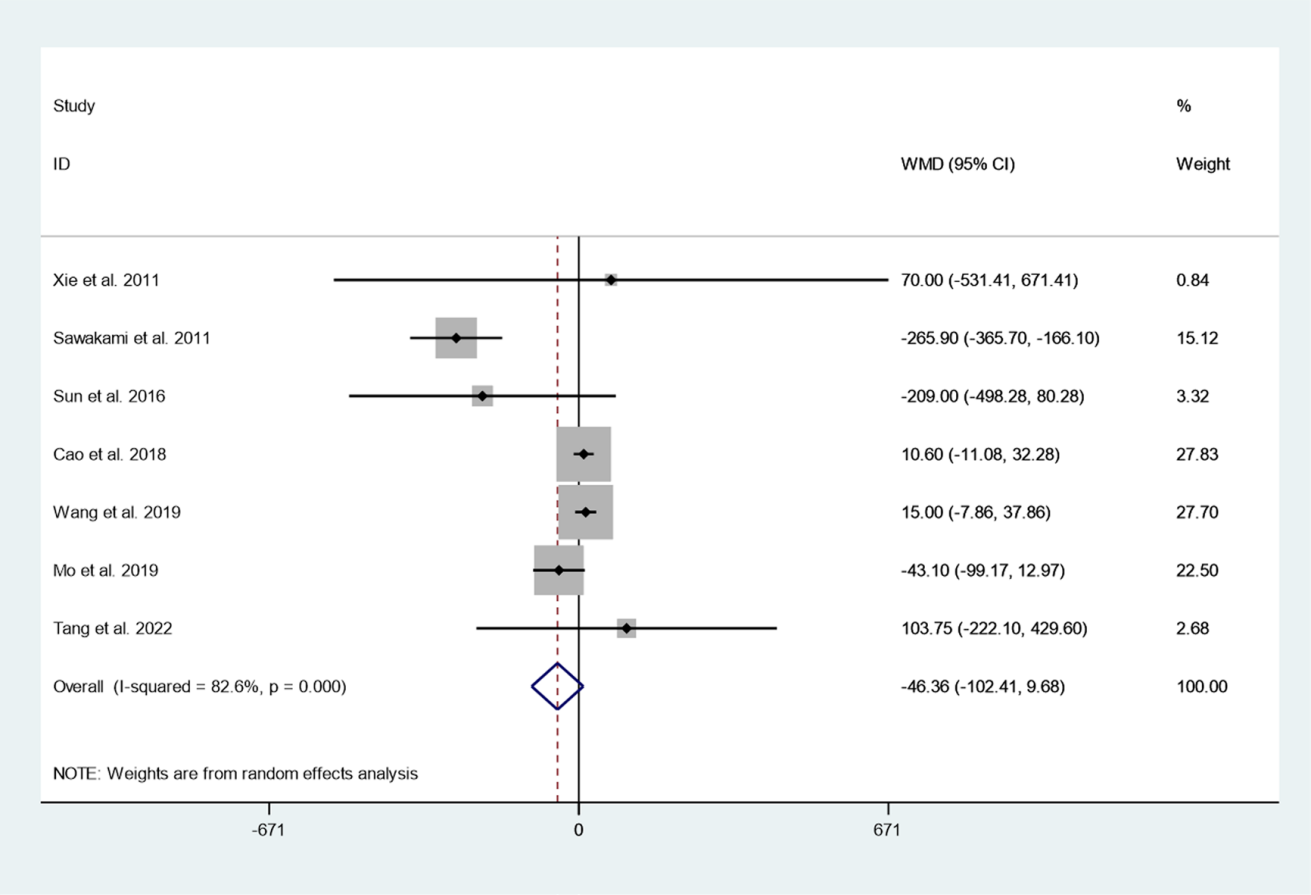
Forest plot of intraoperative blood loss between cement augmented screw group and conventional screw group

Seven articles reported intraoperative blood loss between cement augmented screw group and conventional screw group. Since there was significant heterogeneity in the study, the random-effect model was used to combine the effect sizes (*I*^*2*^ = 82.6%, *P* = 0.000). The pooled results showed that the difference in intraoperative blood loss between the cement augmented screw group and the conventional screw group was not statistically significant (WMD = − 46.36, 95% CI − 102.41 to 9.68; *P* = 0.105) (Fig. 4).

#### JOA score

**Fig. 5:**
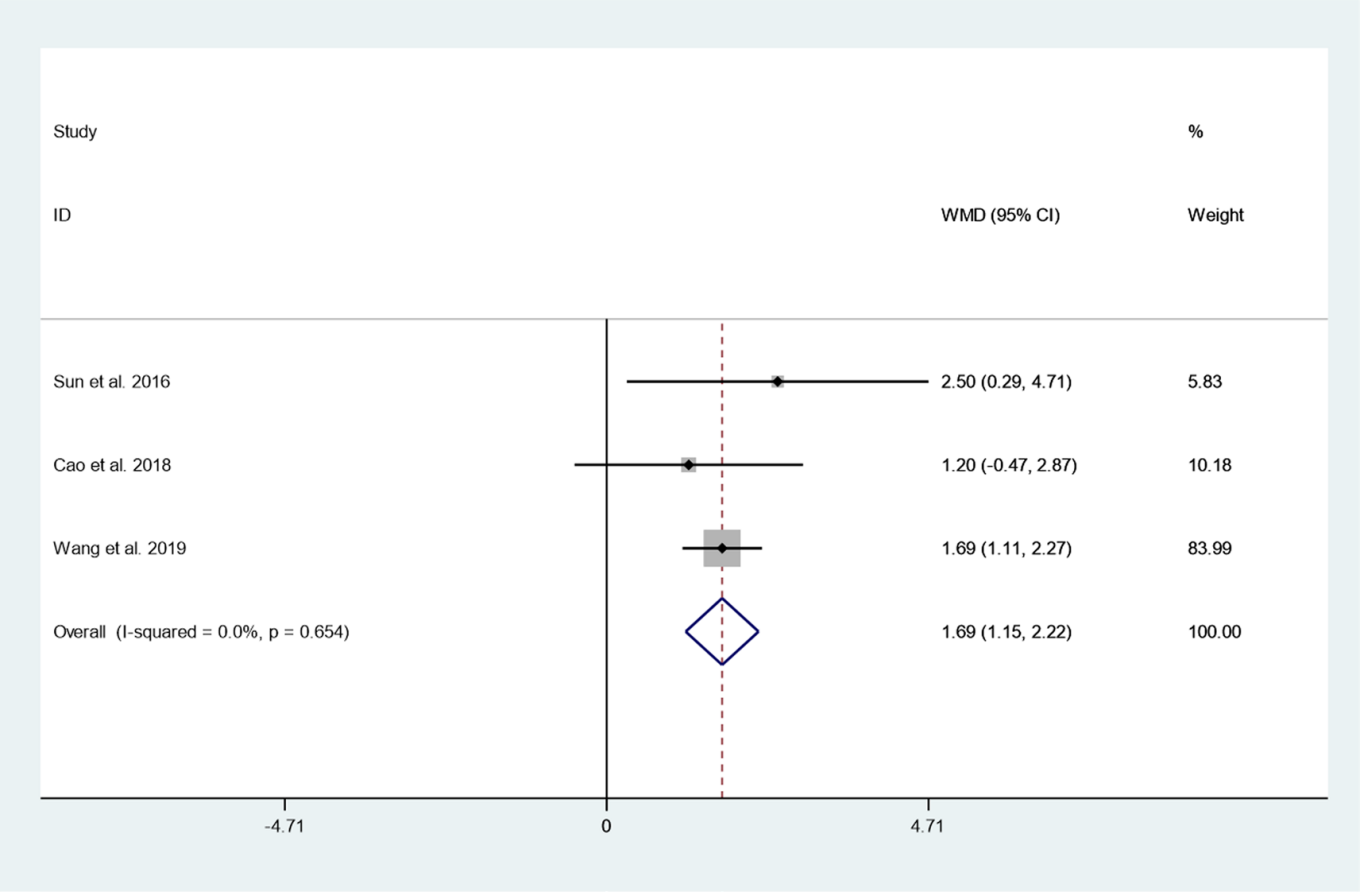
Forest plot of JOA score between cement augmented screw group and conventional screw group

Three articles reported JOA score between cement augmented screw group and conventional screw group. Since there was no heterogeneity in the study, the fixed-effect model was used to combine the effect sizes (*I*^*2*^ = 0.0%, *P* = 0.654). The pooled results show that the JOA score of the cement augmented screw group was significantly higher than that of the conventional screw group (WMD = 1.69, 95% CI 1.15 to 2.22; *P* = 0.000) (Fig. 5).

#### VAS score

**Fig. 6:**
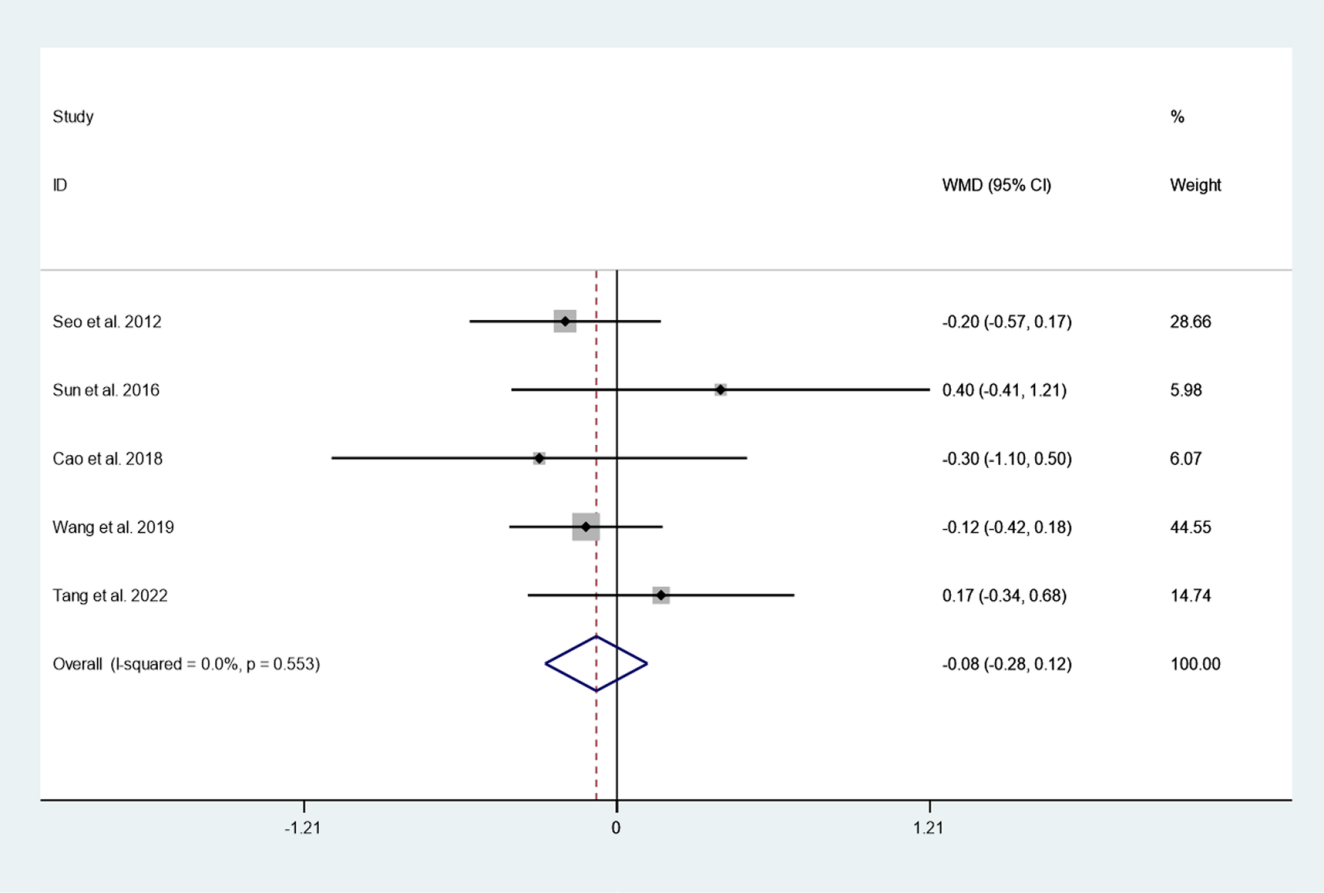
Forest plot of VAS score between cement augmented screw group and conventional screw group

Five articles reported VAS score between cement augmented screw group and conventional screw group. Since there was no heterogeneity in the study, the fixed-effect model was used to combine the effect sizes (*I*^*2*^ = 0.0%, *P* = 0.553). The pooled results showed that the difference in VAS score between the cement augmented screw group and the conventional screw group was not statistically significant (WMD = − 0.08, 95% CI − 0.28 to 0.12; *P* = 0.427) (Fig. 6).

#### Intervertebral space height

**Fig. 7:**
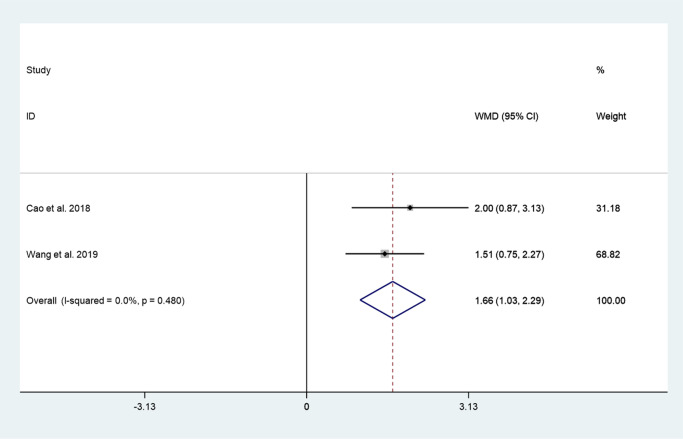
Forest plot of intervertebral space height between cement augmented screw group and conventional screw group

Two articles reported intervertebral space height between cement augmented screw group and conventional screw group. Since there was no heterogeneity in the study, the fixed-effect model was used to combine the effect sizes (*I*^*2*^ = 0.0%, *P* = 0.480). The pooled results show that the intervertebral space height of the cement augmented screw group was significantly higher than that of the conventional screw group (WMD = 1.66, 95% CI 1.03 to 2.29; *P* = 0.000) (Fig. 7).

#### Post-operation fusion rate

**Fig. 8:**
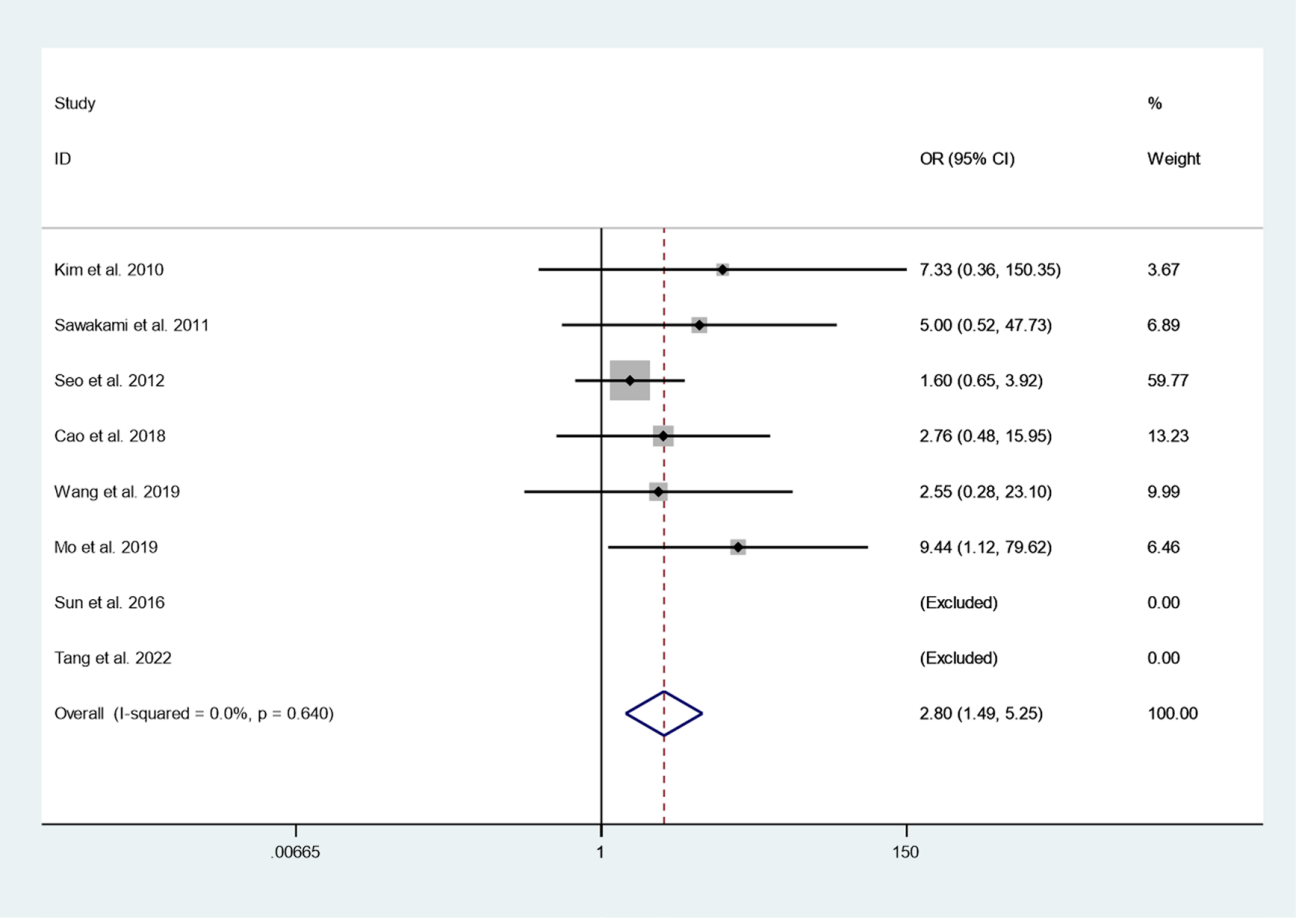
Forest plot of post-operation fusion rate between cement augmented screw group and conventional screw group

Eight articles reported post-operation fusion rate between cement augmented screw group and conventional screw group. Since there was no heterogeneity in the study, the fixed-effect model was used to combine the effect sizes (*I*^*2*^ = 0.0%, *P* = 0.480). The pooled results show that the post-operation fusion rate of the cement augmented screw group was significantly higher than that of the conventional screw group (OR = 2.80, 95% CI 1.49 to 5.25; *P* = 0.001) (Fig. 8).

#### Screw loosening rate

**Fig. 9:**
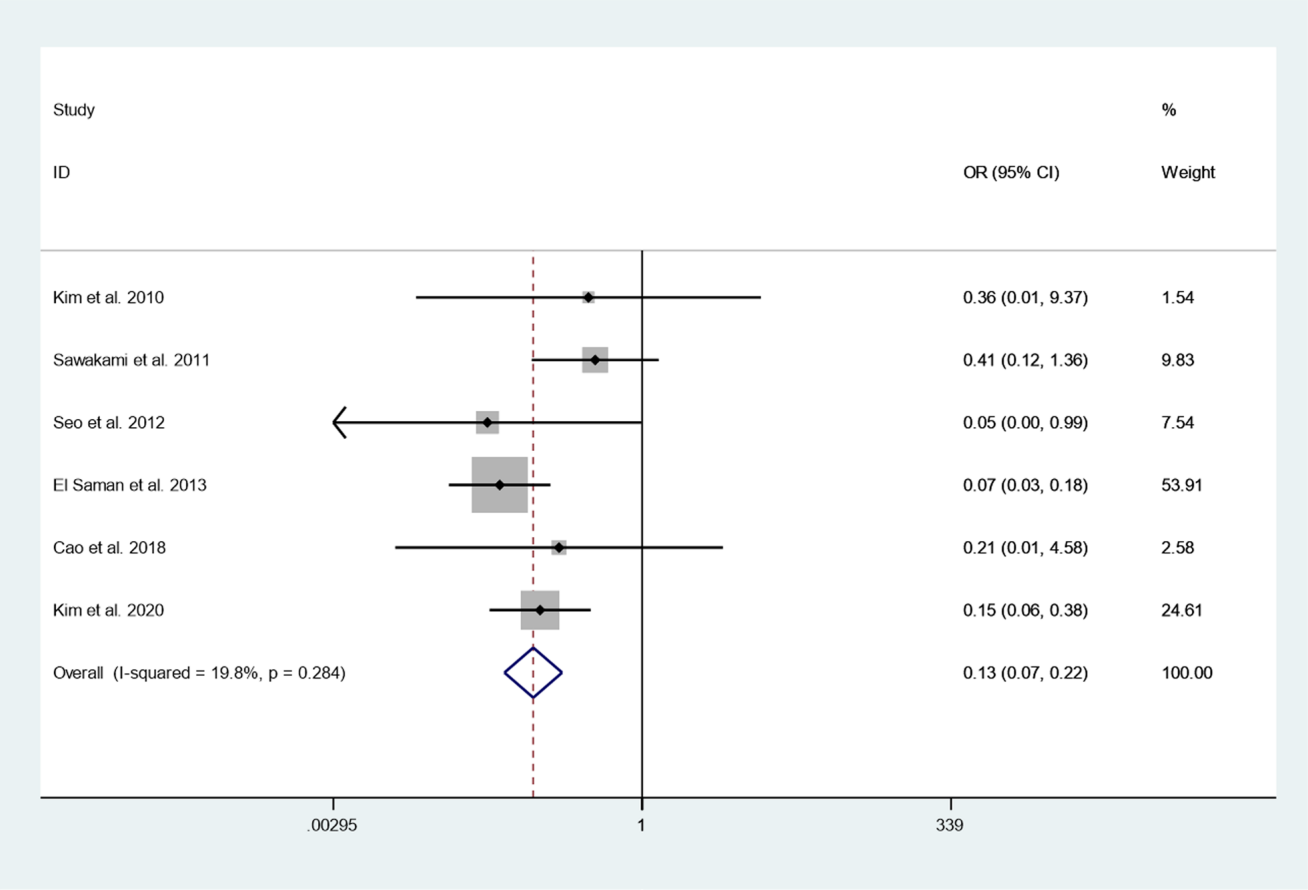
Forest plot of screw loosening rate between cement augmented screw group and conventional screw group

Six articles reported screw loosening rate between cement augmented screw group and conventional screw group. Since there was no heterogeneity in the study, the fixed-effect model was used to combine the effect sizes (*I*^*2*^ = 19.8%, *P* = 0.284). The pooled results show that the screw loosening rate of the cement augmented screw group was significantly lower than that of the conventional screw group (OR = 0.13, 95% CI 0.07 to 0.22; *P* = 0.000) (Fig. 9).

##### Subgroup analysis

We further performed subgroup analyses based on cement strengthening methods.

##### JOA score

**Fig. 10:**
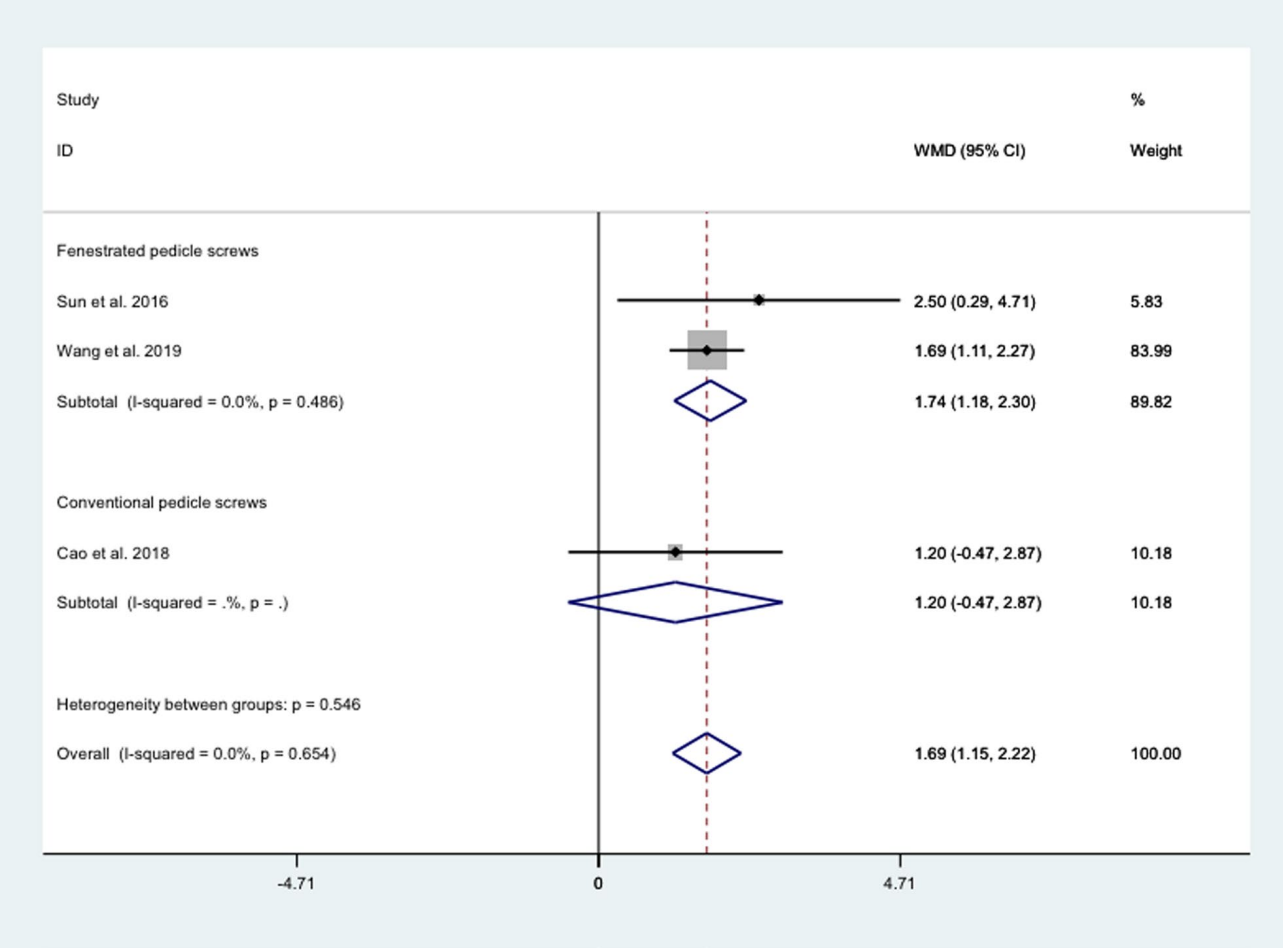
Differences between different cement strengthening modalities and conventional screw therapy in JOA core

The pooled results showed that the difference between cement treatment with conventional pedicle screws and conventional screw treatment in JOA was not statistically significant (WMD = 1.20, 95% CI − 0.47 to 2.87; *P* = 0.159), while the JOA after cement treatment with fenestrated pedicle screws was significantly higher than that of conventional screw treatment (WMD = 1.74, 95% CI 1.18 to 2.30; *P* = 0.000) (Fig. 10).

##### VAS

**Fig. 11:**
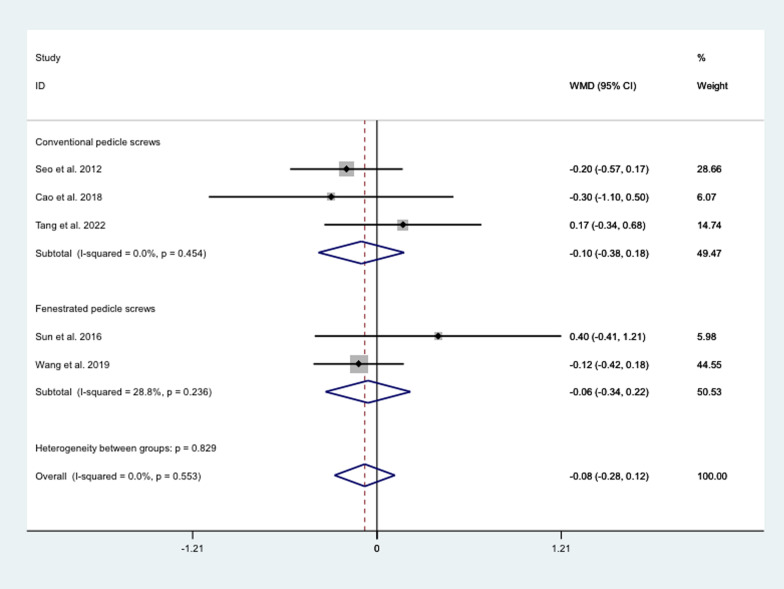
Differences between different cement strengthening modalities and conventional screw therapy in VAS

The pooled results showed that the difference between cement treatment with conventional pedicle screws (WMD = − 0.10, 95% CI − 0.38 to 0.18; *P* = 0.476), or fenestrated pedicle screws (WMD = − 0.06, 95% CI − 0.34 to 0.22; *P* = 0.680) and conventional screw treatment in JOA were all not statistically significant (Fig. 11).

##### Fusion rate

**Fig. 12:**
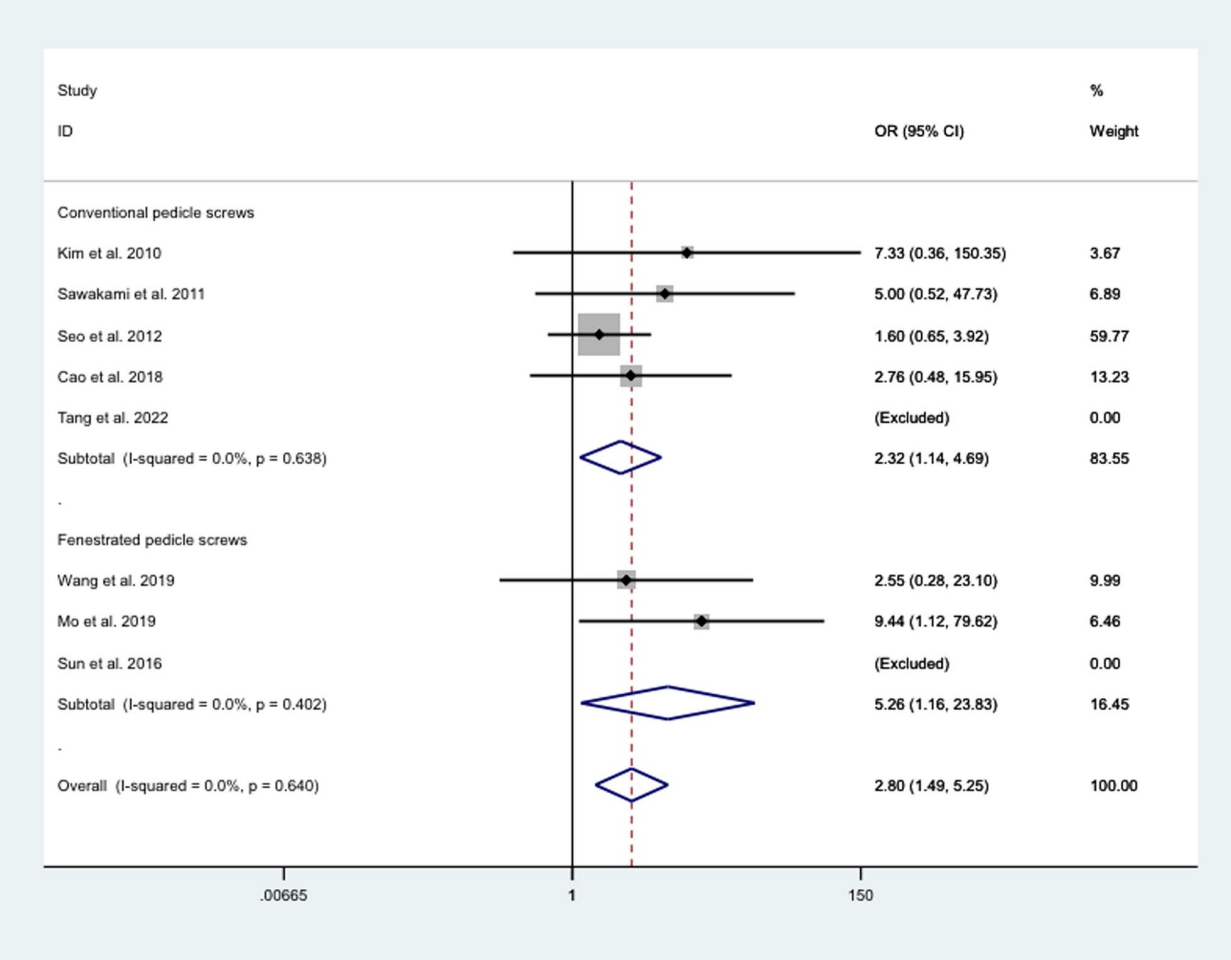
Differences between different cement strengthening modalities and conventional screw therapy in fusion rate

The poole results showed that the fusion rate after bone cement treatment with conventional pedicle screws (OR = 2.32, 95% CI 1.14 to 4.69; *P* = 0.02) or fenestrated pedicle screws (OR = 5.26, 95% CI 1.16 to 23.83; *P* = 0.031) were all significantly higher than that of conventional screw treatment (Fig. 12).

### Sensitivity analysis

The remaining investigations were subjected to a summative analysis to see whether any of the included studies had a disproportionate influence on the meta-overall analysis's results, which was accomplished using sensitivity analyses that eliminated each included research one at a time. According to the meta-analysis, no research had a substantial influence on its results, suggesting that the findings were steady and credible (Additional file 1: Figs. S1–S7).

### Publication bias

**Fig. 13:**
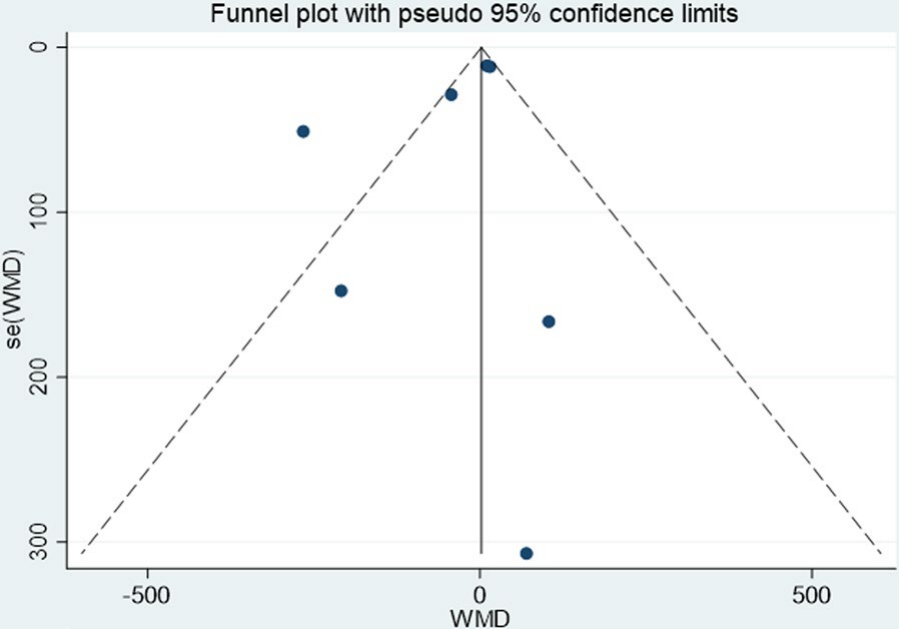
Funnel plot used to assess publication bias

The funnel plot of this study is shown in Fig. 13. It can be seen that the funnel plot was symmetrical, and the *P* value of Egger’s test was 0.242, respective, indicating that there is no obvious publication bias in this study.

## Discussion

The development of medical technology has led to the continuous aging of the population, and the lack of calcium supplements, sedentary and smoking, and the increasing number of the elderly population, leading to the increasing number of people suffering from osteoporosis. In osteoporosis patients with lumbar disc herniation, spinal stenosis, lumbar spondylolisthesis instability and scoliosis, and other degenerative diseases, spinal fusion and fixation with pedicle screw system is one of the main treatment methods [25]. However, patients with osteoporosis are prone to use conventional pedicle screws in the early stage, with pedicle and vertebral fractures, and the late stage may lead to progressive, junction kyphosis, pseudarthrosis formation and degeneration of adjacent vertebral segments, and the most common complication is screw loosening [26]. Studies have shown that spinal fixation stability is only 60% [27] compared to spines with good bone quality (> 120 mg/cm^3^) and below 0.6 g/cm^2^ for early screw loosening [28].

At present, many scholars use a variety of ways and technology efforts to improve the stability of spinal fixation, such as some scholars in the original fixed, on the basis of supplementary anterior fixation, laminar hook and install transverse connector, and more scholars to enhance pedicle screw, pull force, reduce pine, nail and other related complications risk, constantly improve technology [29]. Bone cement has the advantages of short setting time and high curing stability, and is widely used in the treatment of osteoporosis related diseases [30]. The study of ERDEM et al. [31] found that the pull-out force of pedicle screws increased significantly when using bone cement strengthening fixation, with an increase of 96–262%, while the lateral bending stiffness of pedicle screws increased by 153%, so this technique was gradually applied by spinologists to the surgical treatment of patients with degenerative diseases of the thoracolumbar spine [31].

A total of 12 articles were included in this study, and the results of meta-analysis showed that the operation time of the bone cement-strengthened pedicle screw group was more than that of the traditional pedicle screw group, and considering the cement-reinforced pedicle screw group increased the cement injection link, the number of fluoroscopy required also increased significantly, which in turn increased the operation time to a certain extent. There was no difference between the two groups in terms of intraoperative blood loss, hospital stay and postoperative drainage, considering that spinal canal decompression was basically concentrated in spinal canal decompression with spinal surgery bleeding and greater damage to patients, while surgical bleeding and injury were closely related to postoperative recovery, so the recent postoperative recovery speed of the two was similar, resulting in the same length of hospital stay [32].

JOA and VAS are common criteria for evaluating the efficacy of spinal surgery [33]. In this study, the JOA of the cement-enhanced pedicle screw group was better than that of the conventional pedicle screw group, and the follow-up time of the two groups was basically more than 1 year, indicating that the long-term efficacy of the cement-enhanced pedicle screw group was better than that of the conventional pedicle screw, and the treatment of cement-strengthened screw could improve the long-term quality of life of patients.

In addition, in terms of maintaining the height of intervertebral space, improving the fusion rate and reducing the screw loosening rate, the cement-reinforced pedicle screw group was still better than the conventional pedicle screw group. This shows that the use of bone cement strengthening technology can effectively improve the strength of the vertebral body, reduce the risk of loosening and nail retraction, achieve strong internal fixation, reduce the collapse of the final stub, better maintain the height of the intervertebral space, and provide a good and stable mechanical environment for intervertebral fusion, improve the fusion efficiency [34], and achieve the ideal clinical effect.

Furthermore, we performed subgroup analyses based on cement strengthening methods. The summary results suggest that in the analysis of JOA and fusion rate, cement treatment with fenestrated pedicle screws had higher postoperative JOA and fusion rate than cement treatment with conventional pedicle screws, indicating that fenestrated pedicle screws may be a better treatment and could be further promoted in the future.

This meta-analysis has several limitations. First, all of the studies included in this research were cohort studies. The literature quality is lower than that of randomized controlled trials, which may lead to selection bias. In addition, the included sample size was too small, and some studies had large statistical heterogeneity, resulting in reduced the reliability of the analysis results. The conclusions obtained in this paper need to be further verified by more rigorous high-quality, large-sample clinical studies.

## Conclusion

Compared with conventional pedicle screw placement, cement-augmented pedicle screw is more effective in the treatment of osteoporotic thoracolumbar degenerative disease by improving fusion rate and interbody height, reducing the incidence of screw loosening, and elevating long-term efficacy.

### Supplementary Information

**Additional file 1**. Plots of sensitivity analysis.

## Data Availability

The datasets are available from the corresponding author on reasonable request.
