# Supplementary material for: Cement-augmented pedicle screw for thoracolumbar degenerative diseases with osteoporosis: a systematic review and meta-analysis
Source: J Orthop Surg Res. 2023 Aug 28;18:631. doi: 10.1186/s13018-023-04077-w (PMC10464480; doi:10.1186/s13018-023-04077-w)
Supplement: Supplementary file 1 — Additional file 1. Plots of sensitivity analysis. [file 13018_2023_4077_MOESM1_ESM.docx]

Figure S1. Sensitivity analysis of hospitalization time between cementaugmented screw group and conventional screw group

Figure S2. Sensitivity analysis of operation time between cementaugmented screw group and conventional screw group

Figure S3. Sensitivity analysis of intraoperative blood loss between cementaugmented screw group and conventional screw group

Figure S4. Sensitivity analysis of JOA score between cementaugmented screw group and conventional screw group

Figure S5. Sensitivity analysis of VAS score between cementaugmented screw group and conventional screw group

Figure S6. Sensitivity analysis of post-operation fusion rate between cementaugmented screw group and conventional screw group

Figure S7. Sensitivity analysis of screw loosening rate between cementaugmented screw group and conventional screw group
